# MicroRNAs in Lung Cancer Brain Metastasis

**DOI:** 10.3390/ijms251910325

**Published:** 2024-09-25

**Authors:** Israel Martínez-Espinosa, José A. Serrato, Blanca Ortiz-Quintero

**Affiliations:** Department of Molecular Biomedicine and Translational Research, Instituto Nacional de Enfermedades Respiratorias Ismael Cosío Villegas, 14080 Mexico City, Mexico

**Keywords:** MicroRNAs, brain metastasis, lung cancer

## Abstract

Brain metastasis is a significant clinical challenge for patients with advanced lung cancer, occurring in about 20–40% of cases. Brain metastasis causes severe neurological symptoms, leading to a poor prognosis and contributing significantly to lung cancer-related mortality. However, the underlying molecular mechanism behind brain metastasis remains largely unknown. MicroRNAs (miRNAs) are small, non-coding RNAs linked to several aspects of cancer progression, including metastasis. In the context of lung cancer, significant research has shown the involvement of miRNAs in regulating critical pathways related to metastatic spread to the brain. This review summarizes the scientific evidence regarding the regulatory roles of intra- and extracellular miRNAs, which specifically drive the spread of lung cancer cells to the brain. It also revises the known molecular mechanisms of brain metastasis, focusing on those from lung cancer as the primary tumor to better understand the complex mechanisms underlying this regulation. Understanding these complex regulatory mechanisms holds promise for developing novel diagnostic biomarkers and potential therapeutic strategies in brain metastasis.

## 1. Introduction

Lung cancer has the highest incidence of primary tumors that spread to the brain, followed by breast cancer and melanoma [1,2,3]. Brain metastasis is found in around 15–20% of patients with non-small cell lung cancer (NSCLC) at the time of diagnosis, and this number may increase to 25–40% during the disease [4,5,6]. Patients with advanced-stage IV NSCLC and the histological subtype adenocarcinoma have a higher incidence of brain metastasis at the time of diagnosis, with numbers rising to 40–50% [7,8,9]. Among NSCLC patients, those with EGFR/ALK mutations show a higher prevalence and incidence of brain metastasis [10]. The incidence of brain metastasis is also high in small cell lung cancer (SCLC), affecting around 10–20% of patients at their initial diagnosis [11]. Lung cancer patients with brain metastasis have a poor prognosis. Early studies showed a median survival rate of a few months to around a year after diagnosis, which drops to less than six months if left untreated [12,13]. However, the survival rate has improved due to advancements in molecular targeted therapies and immunotherapies, combined with the graded prognostic assessment for lung cancer using molecular markers (Lung-molGPA) index for predicting prognosis. Currently, patients with adenocarcinoma and brain metastasis have a median survival time of around 15 months [14]. Those with a Lung-molGPA score of 3.5 to 4 can have a median survival time of about four years, but only 4% of patients achieve these high scores [15]. The Lung-molGPA index predicts brain metastasis prognosis based on various factors, including age, performance, number of metastases, extracranial disease, and genetic alterations such as EGFR and ALK mutations [15]. Therefore, significant efforts have been made to predict outcomes for patients with brain metastasis and determine the best treatment options. However, there is still a limited understanding of the complex pathogenic mechanisms driving brain metastasis, which has become a significant obstacle to finding better therapies. Although some major steps of the brain metastatic cascade have been identified, the complete metastasis process is still unclear.

MicroRNAs (miRNAs) regulate various biological processes, including cancer progression and metastasis [16]. These small non-coding RNAs are typically 18–22 nucleotides in length and regulate gene expression post-transcriptionally by binding to the 3′ untranslated region (UTR) of target mRNAs. This binding leads to mRNA degradation or translational repression [17]. In the context of brain metastasis derived from lung cancer, significant research has shown the involvement of miRNAs in regulating critical pathways related to metastatic spread to the brain. Evidence points to miRNAs association with lung cancer brain metastasis by affecting brain blood barrier (BBB) permeability, tumor cell migration, proliferation, invasion, and epithelial–mesenchymal transition (EMT).

This review focuses on the regulatory mechanisms involving those miRNAs that specifically drive the spread of lung cancer cells to the brain. It also revises the known molecular mechanisms of brain metastasis, specifically from lung cancer as the primary tumor, to better understand the complex mechanisms underlying this regulation.

## 2. Mechanisms of Brain Metastasis

### 2.1. General Mechanisms of Metastasis to the Brain

Several studies have provided insights into the complex mechanisms underlying cancer cells spreading to the brain. Like all metastases, this process occurs in multiple stages, beginning with the detachment of tumor cells from the primary site, migration, invasion of nearby stroma, and entry into the bloodstream or lymphatic vessels [18]. Once in circulation, circulating tumor cells (CTCs) face several challenges, including strong mechanical flow, immunosurveillance, and oxidative stress [19,20]. After surviving these challenges, CTCs must extravasate through the blood–brain barrier (BBB) and infiltrate the central nervous system (CNS) to cause brain metastasis [18]. The BBB comprises specialized endothelial cells lining the capillaries in the brain. These brain microvascular endothelial cells (BMECs) and their basement membrane form the walls of the brain blood microvessels. BMECs are firmly attached by adherens and tight junctions, creating a highly selective barrier. BMECs are surrounded by pericytes and astrocytes, contributing to the BBB’s structural integrity and barrier properties. The BMECs, basement membrane, pericytes, astrocytes, neurons, and microglia form the neurovascular unit (NVU), which regulates BBB formation. The BBB is now considered a component of the NVU [21]. The BBB maintains the brain’s homeostasis by tightly controlling the passage of ions, nutrients, and biomolecules while restricting the entry of potentially harmful substances, pathogens, and immune cells from the bloodstream [22,23]. Therefore, the extravasation of CTCs through the BBB is the first delimited and crucial step in developing brain metastases. After reaching the brain, metastatic cancer cells must adapt, survive, and create favorable conditions for colonization. This process relies on intricate interactions with the brain’s microenvironment and the cancer cells’ ability to reach, survive, and multiply efficiently in this organ. Therefore, the second critical step involves adapting and colonizing tumor cells in the brain. The following sections provide a focused overview of the molecular mechanisms of brain metastasis from lung cancer. For more information, additional published reviews offer more detailed insights [18,24].

### 2.2. The Blood Brain Barrier (BBB) Components

The BMECs can uniquely regulate the exchange of ions, various molecules, and cells from the blood into the brain [21,25]. BMECs, their tight junctions, and the basement membrane provide a physical barrier and are critical for the integrity and function of the BBB. The tight junctions between BMECs limit the passage of solutes between cells. Simultaneously, BMECs have low transcytosis rates that restrict the vesicle-mediated movement of solutes across the cells. BMECs express efflux and specific nutrient transporters to control the transfer of lipophilic molecules and nutrients through the BBB. They also express low levels of leucocyte adhesion molecules (LAMs) to limit the entrance of immune cells into the brain. The basement membrane is composed of the inner vascular basement and outer parenchymal basement membranes, consisting of collagen type IV, heparan sulfate proteoglycans, laminin, fibronectin, and other extracellular matrix proteins. The basement membranes provide structural support, an additional barrier for molecules and cells, and anchorage for multiple signaling pathways between the endothelial cells [21,25]. Pericytes are cells that surround brain microvessels and capillaries. They are located near astrocytes and neurons, extending membrane prolongations that partially cover the surface of BMECs. Pericytes regulate intercellular signals, BBB development, cerebral blood flow, infiltration of immune cells, and tissue survival. These cells secrete growth factors that support endothelial cells’ survival and tight junction formation, such as platelet-derived growth factor-B (PDGF-B) and transforming growth factor-beta (TGF-β) [21,26]. Astrocytes are glial cells that almost entirely ensheath the blood vessels using their endfeet protrusions while interacting with BMECs, pericytes, and neurons. Astrocytes are critical in maintaining the integrity and function of the blood–brain barrier (BBB), innate immunity, and cerebrovascular regulation. They secrete regulating factors such as vascular endothelial growth factor (VEGF), transforming growth factor-β (TGFβ), and angiopoietin 1 (ANG1), which promote BMEC function and BBB integrity [22,27].

### 2.3. Extravasation of Lung Cancer Cells through the BBB in Brain Metastasis

The extravasation of tumor cells starts when CTCs are trapped within the capillaries near the brain endothelium due to the slower blood flow and their size. This allows them to roll over and subsequently adhere to the BMECs via multiple receptor–ligand interactions. Then, tumor cells must induce changes in the BBB to cross the barrier and access the brain.

Metastatic NSCLC cells with higher expressions of activated leukocyte cell adhesion molecule (ALCAM), disintegrin metalloproteinase domain-containing protein 9 (ADAM9), CD15, and CD15s (sLex) adhere more strongly to brain endothelial cells during extravasation. Moreover, increased ALCAM and ADAM9 promote brain metastasis in vivo, while the overexpression of CD15 and CD15s disrupts brain endothelial cell monolayers in vitro [28,29,30,31].

On the other hand, decreasing the expression of the major facilitator superfamily domain 2a (Mfsd2a) in brain endothelium leads to increased BBB leakage in a model of lung, breast, and melanoma patient-derived xenograft of BBB [32]. Notably, decreased Mfsd2a expression was observed in vascular endothelial cells within brain metastases from primary lung cancer [32]. Conversely, increasing the expression of Claudin-5 (CLDN5) in brain endothelial cells can reduce paracellular permeability and the migration of lung adenocarcinoma A549 cells across the BBB [33]. Also, BBB function is safeguarded by activating the adenosine A2A receptor on lung adenocarcinoma cells. In addition, it reduces brain metastasis via the stromal cell-derived factor 1/CXC motif chemokine receptor 4 (SDF-1/CXCR4) axis [34]. Another study reported that the Aldo-keto reductase family 1 B10 (AKR1B10), a NADPH-dependent enzyme, was increased in lung cancer brain metastasis. When AKR1B10 is silenced in lung adenocarcinoma cells, it suppresses their extravasation through the BBB in vitro, ex vivo microfluidic chip, and in vivo models; moreover, AKR1B10 silencing induced the downregulation of metastatic cells’ expression of matrix metalloproteinase MMP-2 and MMP-9 [35]. In brain metastasis, dysfunction of the BBB has been linked to the increased expression of matrix metalloproteinases (MMPs), which can degrade tight junctions and basement membrane proteins [36].

Other studies have reported that SCLC cells release factors, such as placental growth factor (PLGF), Visfatin, and Annexin A1, which promote transmigration across the BBB. PGLF, Visfatin, and Annexin A1 are elevated in the serum of SCLC patients with brain metastasis and SCLC cell lines co-cultured with brain endothelial cells [37,38,39]. PLGF derived from SCLC cell lines triggers the activation of vascular endothelial growth factor (VEGF) receptor-1-Rho-extracellular regulated protein kinase 1/2 signaling axis, which promotes the disassembly of tight junctions in brain endothelial cells and tumor cells transmigration in vitro [37]. Meanwhile, Visfatin promotes the transmigration of SCLC cell lines in vitro and induces the CC chemokine ligand 2 (CCL2) expression on tumor cells [38]. The blockage and induced inhibition of annexin A1 inhibit cell adhesion to brain endothelium, transendothelial migration, and metastasis to mice brains [39].

### 2.4. Colonization of Lung Cancer Cells in the Brain

Evidence indicates that brain colonization is highly inefficient due to natural defenses such as reactive astrocytes, microglia, and immune cells surrounding the tumoral cells. However, some cancer cells evade the immune response and remain in the perivascular niche, where they can access nutrients and growth factors [40,41].

Several studies indicate that metastatic brain colonization by lung cancer cells involves intrinsic tumor cell properties and their interactions with the brain microenvironment. These complex interactions allow selected tumor cells to adapt and survive within the brain. Although this process is not fully understood, studies have revealed some relevant mechanisms that facilitate brain metastasis of lung cancer cells. A recent study found that overexpression of PMS1 Homolog 2 (PMS2) in lung cancer cells augments their ability to survive, proliferate, and stimulates the formation of colonies within the brain. In this study, PMS2 amplification was identified in the cerebrospinal fluid samples of lung cancer patients with brain metastases. PMS2 encodes proteins that are crucial in repairing errors during DNA replication. However, the exact mechanism implicated in brain metastasis promotion still needs to be explored [42]. Another study found increased Mucin 5ac (MUC5AC) levels in lung adenocarcinoma brain metastases tissue and brain-tropic cell lines. When MUC5AC is reduced in brain-tropic cells, it decreases brain metastasis and tumor growth, directly affecting the metastasis process. This research discovered that MUC5AC interacts with Annexin A2. Annexin A2 triggers downstream matrix metalloprotease activation and leads to extracellular matrix degradation. Additionally, the expression of MUC5AC was increased by adding an astrocyte-conditioned medium or the chemokine ligand 2 (CCL2), which enhances brain colonization in vivo, highlighting the involvement of astrocytes in the colonization [43].

A recent study discovered the increased expression of heat shock protein 47 (HSP47) in brain metastasis tissue from both human breast and lung cancer. In murine lung tumor cells, the upregulation of HSP47 promotes brain colonization and tumor growth by creating an immune-suppressed environment. HSP47-mediated collagen deposition promotes microglial polarization to the M2 phenotype, increasing anti-inflammatory cytokines and decreasing CD8+ T cell anti-tumor responses [44].

Another mechanism involves cancer cells invading the brain and evading the immune system. Brain metastatic cells derived from breast and lung cancer secrete plasminogen activator (PA) inhibitory serpins, which protect against the loss of cell adhesion capacity caused by L1 cell adhesion molecule (L1CAM) degradation and against Fas-dependent apoptosis mediated by reactive astrocytes [45].

Table 1 summarizes the known molecular factors associated with mechanisms of brain metastases in lung cancer.

**Table 1:** Molecular factors associated with mechanisms of brain metastases in lung cancer.

Factor	Expression	Source	Effect and Mechanism	Step of the Brain Metastasis Process	Reference
ALCAM and ADAM9	Overexpression	Metastatic NSCLC cells	Higher adhesion to brain endothelial cells. Promote brain metastasis in vivo.	Adhesion to brain endothelial cells	[28,29]
CD15, and CD15s (sLex),	Overexpression	NSCLC cells	Higher adhesion to brain endothelial cells.	Adhesion to brain endothelial cells	[31]
CLDN5	Induced overexpression	BMECs	Reduce paracellular permeability and the migration of A549 in vitro.	Alteration of BBB permeability	[33]
Mfsd2a	Downregulation	BMECs within brain metastases from primary lung cancer	Increase BBB leakage.	Alteration of BBB permeability	[32]
Adenosine A2A receptor	Induced activation	Lung adenocarcinoma cells	Protect the BBB function and reduces brain metastasis via SDF-1/CXCR4 axis in vitro	Disruption of BBB	[34]
AKR1B10	Overexpression	Brain metastatic tissue from primary lung cancer.	Silencing AKR1B10 in lung adenocarcinoma cells suppresses extravasation in vitro, ex vivo, and in vivo; and downregulate MMP-2 and MMP-9 expression	Extravasation, disruption of BBB	[35]
PLGF	Increased secretion and expression	SCLC cells cocultured with BMECs. Serum of SCLC patients.	Disassembly of tight junctions in brain endothelial cells and promotion of tumor cells transmigration in vitro, via activation of VEGF receptor-1-Rho-ERK1/2 signaling axis.	Transendothelial migration	[37]
Visfatin	Increased secretion and expression	SCLC cells cocultured with BMECs. Serum of SCLC patients.	Promotes the transmigration in vitro.	Transendothelial migration	[38]
Annexin A1	Increased secretion and expression	SCLC cells cocultured with BMECs. Serum of SCLC patients.	Blocking Annexin A1 inhibited adhesion to brain endothelial cells, transendothelial migration, and metastasis to mice brain.	Adhesion, transendothelial migration, and metastasis to brain	[39]
PMS2	Gene Amplification. Induced overexpression.	CSF of lung cancer patients with brain metastases. Lung cancer cells.	Increased metastatic potential and establish colonies within the brain.	Brain colonization	[42]
CCL2	Increased secretion	Reactive astrocytes	Increase MUC5AC expression in lung cancer cells and brain colonization	Brain colonization	[43]
HSP47	Overexpression	Brain metastatic tissue from primary breast and lung adenocarcinoma	Increased brain colonization by creating an immune-suppressed environment, via microglial polarization to M2 and reduced CD8+ T cell response.	Brain colonization	[44]
Serpins	Overexpression	Brain metastatic cells from breast and lung cancer	Protection against Fas-dependent apoptosis mediated by astrocytes and against loss of cell adhesion capacity by L1CAM degradation	Brain colonization	[45]

NSCLC, non-small cell lung cancer; SCLC, small cell lung cancer; CSF, cerebrospinal fluid; BMECs, brain microvascular endothelial cells; ALCAM, activated leukocyte cell adhesion molecule; ADAM9, disintegrin metalloproteinase domain-containing protein 9; CLDN5, Claudin-5; Mfsd2a, Major facilitator superfamily domain 2a; AKR1B10, aldo-keto reductase family 1 B10; PLGF, placental growth factor; PMS2, PMS1 Homolog 2; CCL2, CC chemokine ligand 2; HSP47, heat shock protein 47; SDF-1/CXCR4, stromal cell-derived factor 1/CXC motif chemokine receptor 4; MUC5AC, Mucin 5ac; MMP, matrix metalloproteinase; L1CAM, L1 cell adhesion molecule.

Recently, researchers discovered that cancer cells undergo significant metabolic changes to adapt to the unique microenvironment of the brain. These changes are influenced by the brain’s specific metabolic needs, which vary depending on the location of the metastasis within the brain. For example, the brain parenchyma has high energy supply and consumption but low energy reserves. In contrast, the subarachnoid space lacks oxygen and nutrients and is filled by cerebrospinal fluid [46,47]. Metastases in the parenchyma are the most common, although up to 10% of cancer patients experience metastasis in the subarachnoid space, called leptomeningeal metastasis. Currently, most of the evidence of metabolic adaptation comes from brain metastasis resulting from breast cancer and melanoma [46,48,49,50]. However, there is a lack of data on metabolic adaptation in lung cancer brain metastasis. For instance, breast cancer cells undergoing brain metastasis increase activity in energy production pathways like glycolysis, tricarboxylic acid cycle, and oxidative phosphorylation. They also activate the pentose phosphate pathway and the glutathione system to reduce reactive oxygen species from the increased oxidative metabolism [51]. In another example, fatty acid-binding protein 7 (FABP7), a brain-specific intracellular lipid-binding protein, promotes the metabolic reprogramming of metastatic HER2+ breast cancer cells [52].

## 3. MicroRNAs

MicroRNAs (miRNAs) are small, non-coding RNA molecules of approximately 22 nucleotides in length. They regulate gene expression post-transcriptionally by inhibiting the translation of target messenger RNAs (mRNAs). Under typical physiological conditions, miRNAs control several cellular functions, including angiogenesis, differentiation, cell cycle progression, and apoptosis. The function of miRNAs is also crucial for the proper development of various organs and systems, including the heart, intestine, central nervous system, and respiratory system. In pathological conditions such as cancer, miRNAs exhibit altered expression patterns associated with cancer progression and metastasis [16,17,53,54].

Most conserved miRNAs among metazoan are generated through a canonical biogenesis pathway [17,54]. In this pathway, the RNA polymerase II (Pol II) transcribes miRNA genes into long primary precursors (pri-miRNAs) [55,56]. The Pri-miRNA is then cleaved by a Micro-processor complex, producing a precursor miRNA (pre-miRNA) hairpin of 55–70 nucleotides in length. This pre-miRNA is then exported to the cytoplasm [57], where the RNA III Dicer/TRBP complex cleaves the pre-miRNA to produce a double-stranded mature miRNA approximately 22 nucleotides long. The mature miRNA is loaded into argonaute 2 (AGO2) with the assistance of Dicer/TRBP and other proteins to form the RNA-induced silencing complex (RISC) [58,59]. One strand of the miRNA (guide strand) is retained on RISC, while the other (passenger strand) is expelled. The guide strand binds to a partially complementary sequence in the target mRNA’s 3′ untranslated region (UTR) [60,61]. miRNA-mRNA binding leads to the repression of target transcripts via mRNA decay and translational repression [53,62,63]. The repression of target transcripts by miRNAs is also known as gene silencing [53].

In addition to their intracellular function, cells release miRNAs into the extracellular space through a highly regulated process involving extracellular vesicles (EVs). MiRNAs and other biologically active molecules, such as proteins, DNA, other nucleic acids, and lipids, are transported as cargo inside vesicles. Cells deliver EVs into the extracellular space, where neighboring recipient cells can take them in, transferring their cargo. The extracellular miRNAs carried by EVs have significant functional implications in recipient cells. Once within the recipient cells, miRNAs bind to complementary sequences in target mRNAs to inhibit translation or cause mRNA decay. This is how they can change the expression of genes. Exosomes and microvesicles are the main types of EVs that transport miRNAs between cells. However, most research has focused on the role of miRNAs transported in exosomes, particularly in the cancer field [64,65,66]. MiRNAs that function intracellularly are also called endogenous miRNAs, while extracellular miRNAs transported in exosomes are known as exosomal miRNAs. Notably, exosomal miRNAs can reach recipient cells in distant organs and tissues by traveling through circulation and recognizing specific molecules and receptors on their target cells. These extracellular miRNAs are found in various body fluids, such as serum, plasma, urine, tears, and saliva. Their levels can indicate pathological conditions such as cancer, offering a potentially less invasive method for diagnosing, monitoring, and predicting cancer as biomarkers [53].

## 4. Role of miRNAs in Lung Cancer Brain Metastasis

The current literature indicates that endogenous miRNAs play a crucial role in the progression of brain metastasis in lung cancer, particularly in NSCLC. They are implicated in mechanisms such as extravasation, the dysregulation of BBB, EMT, migration, colonization, stemness regulation, and creating a supportive microenvironment for brain metastasis. Furthermore, miRNAs have the potential to serve as biomarkers for the diagnosis and prognosis of brain metastasis in lung cancer. However, there is a notable research gap regarding the influence of exosomal miRNAs on the advancement of brain metastasis in lung cancer. Additionally, other non-coding RNAs significantly impact brain metastasis in lung cancer. Further published articles provide more information on this topic [67]. Similarly, other published articles contain information about the miRNAs involved in brain metastasis from breast cancer and melanoma [68,69].

### 4.1. miRNAs as Biomarkers

Some studies have identified specific miRNAs whose altered expression levels are significantly associated with the presence of brain metastasis and lower survival rates in lung cancer patients. These miRNAs, including miR-21, miR-184, miR-197, miR-199, and miR-375, are either overexpressed or underexpressed in lung cancer with brain metastasis. They are potential biomarkers for brain metastasis diagnosis or cancer prognosis (Table 2). For instance, a study found that miR-21 levels in the serum of NSCLC patients with brain metastasis were significantly higher than in those without brain metastasis. An analysis of 26 patients who later developed brain metastasis out of a total of 64 patients showed that the high expression of miR-21 can predict the development of brain metastasis with an AUC of 0.873, a sensitivity of 92.3%, a specificity of 60.7%, and a cutoff point of 0.205. In this study, patients with a high expression of miR-21 experienced brain metastasis 17.4 months after the initial assessment [70]. In another study, levels of miR-184 and miRNA-197 were significantly higher in the tumor tissue of NSCLC patients with EGFR mutation and brain metastasis than those without brain metastasis. However, the current study did not provide strong statistical analysis to support these miRNAs as reliable risk biomarkers for brain metastasis [71]. On the other hand, levels of miR-375 were significantly lower in lung tissue and metastatic brain tissue of NSCLC patients with brain metastasis compared with those without brain metastasis. Statistical analysis showed that decreased levels of miR-375 correlated with advanced disease stage and shorter overall survival (HR = 5.48, 95% CI: 1.93–15.56, *p* = 0.001) [72]. On the other hand, miRNA signatures can also serve as biomarkers for brain metastasis in NSCLC. When analyzing tissue biopsies from primary NSCLC and brain metastasis, researchers found a set of 11 microRNAs with differential expression levels, each with potential diagnostic value for brain metastasis. Five miRNAs (miR-129-2-3p, miR-124-3p, miR-219a-2-3p, miR-219a-5p, and miR-9-5p) were upregulated, while six miRNAs (miR-142-3p, miR-150-5p, miR-199b-5p, miR-199a-3p, miR-199b-3p, and miR-199a-5p) were downregulated in brain metastasis. Among these miRNAs, the miR-199 family showed the best diagnostic value (AUC > 0.92) [73]. Another study identified a signature of three miRNAs, miR-214, miR-210 (upregulated), and miR-15a (downregulated), in lung tissue of lung adenocarcinoma patients with brain metastasis compared to those without brain metastasis. This three-miRNA signature demonstrated strong diagnostic capability for brain metastasis in lung adenocarcinoma patients (AUC = 0.913) [74].

Noticeably, there are currently a few potential miRNA biomarkers for brain metastasis in lung cancer patients, especially when it is compared with lung cancer in general. Most of these biomarkers are found in tissue, which is not ideal as lung or brain tissue samples would be required. A valuable diagnostic or prognostic marker for lung cancer brain metastasis would be a noninvasive or minimally invasive miRNA obtained from body fluids, such as peripheral blood. In contrast, several miRNAs found in blood and other fluids have been identified as potential biomarkers for diagnosis, prognosis, and therapy response in lung cancer, particularly NSCLC [75]. In addition, few studies have investigated the potential diagnostic value of miRNAs for predicting brain metastasis in patients with lung cancer. One study indicated that high miR-21 expression in serum could predict the occurrence of brain metastasis approximately 17.4 months after initial assessment [70]. Another study found that higher levels of miR-330-3p in serum were associated with a shorter time to developing brain metastasis, with a follow-up period of around 20 months [76]. Conducting more longitudinal studies will provide valuable insights into whether specific miRNA expression levels can predict the occurrence or predisposition to brain metastasis in patients with lung cancer.

In Table 2, we used the term “biomarker” to indicate that, based on the references, specific miRNAs could potentially serve as biomarkers for lung cancer brain metastasis.

**Table 2:** MicroRNAs with dysregulated expression associated with brain metastasis in lung cancer patients.

MicroRNA	Expression	Source	Effect	Function	Reference
miR-9	Up	Irradiation-induced M1-type microglia	Irradiation-induced M1-type microglia inhibits MET of NSCLC cells via miR-9/CDH1 Axis	TS miRNA	[77]
miR-21	Up	Serum of NSCLC patients with brain metastasis	Induced overexpression promotes NSCLC cell migration, invasion, proliferation, and angiogenesis.	OncomiR. Predictive biomarker in serum	[70]
miR-21	Up	Lung tissue of cancer patients	Induced overexpression increases brain metastasis initiating cells (BMIC) self-renewal and proliferation.	OncomiR	[78]
miR-184 and miR-197	Up	EGFR-mutant lung tissue of NSCLC patients with brain metastasis	Undetermined	Potential Biomarker in tissue.	[71]
miR-328-5p	Up	Lung tissue of NSCLC patients with brain metastasis	Induced overexpression promotes A549 cell migration.	OncomiR	[79]
miR-378	Up	Lung and brain tissue of NSCLC patients with brain metastasis	Induced overexpression promotes cell survival, migration, and invasion of A549, and vasculogenic mimicry.	OncomiR	[80]
Exosomal miR-550a-3-5p	Up	Plasma of NSCLC and brain metastatic NSCLC cell line.	Induced overexpression increases cell viability, apoptosis, cell cycle, and migration of BMECs via YAP1	OncomiR	[81]
miR-193b	Down	Brain metastatic lung cancer cell line	Induced overexpression inhibits cell invasion and migration.	TS miRNA	[82]
miR-199a-3p/5p, and miR-199b-5p	Down	Brain metastatic tissue from lung cancer patients	Panel of 11 miRNAs with diagnostic value in brain metastasis vs. primary NSCLC. The miR-199 family displayed the highest diagnostic potential.	Biomarker in tissue	[73]
miR-215-3p	Down	Brain metastasis tissue from AD cancer patients	Induced overexpression reduces cell migration and invasion of NSCLC cells.	TS miRNA	[83]
miR-217	Down	Lung adenocarcinoma brain metastasis cell line PC-14/B.	Induced overexpression inhibits cell invasion, proliferation, and migration, via targeting SIRT1 and P53/KAI1 Signaling	TS miRNA	[84]
miR-375	Down	Lung tissue of NSCLC patients with brain metastasis and metastatic brain tissue	Decreased miR-375 correlated with advanced disease stage and shorter overall survival	Biomarker in tissue	[72]
miR-768-3p	Down	Co-cultures lung cancer cells with astrocytes. Brain metastatic tissue from lung cancer patients.	Downregulation enhances cell viability and K-ras expression	TS miRNA	[85]
miR-210, miR-214, y miR-15a	Signature	Lung tissue of AD patients with brain metastasis	Three-miRNA signature predicts the brain metastasis of AD patients	Biomarker in tissue	[74]

TS, tumor suppressor; NSCLC, non-small cell lung cancer; EGFR, epidermal growth factor receptor; BMECs, brain microvascular endothelial cells; YAP1, yes-associated protein 1; AD, lung adenocarcinoma; SIRT1, sirtuin (silent mating type information regulation 2-homolog) 1.

### 4.2. miRNAs Acting as OncomiRs or Tumor Suppressor (TS) MiRNAs

Besides being potential biomarkers, dysregulated miRNAs can act as oncogenic (oncomiRs) or tumor suppressor (TS) miRNAs, depending on their expression context and the target genes they regulate. Oncogenic miRNAs, or oncomiRs, promote carcinogenesis and tumor progression by targeting tumor suppressor genes and critical pathways associated with cancer suppression. Conversely, TS miRNAs inhibit the expression of genes that promote oncogenesis, thus suppressing tumor formation. OncomiRs are usually upregulated in cancer cells, and TS miRNAs are downregulated. In lung cancer patients with brain metastasis, upregulated miRNAs such as miR-21, miR-328, miR-378, miR-550a, and miR-378 are associated with mechanisms promoting metastasis as oncomiRs. On the other hand, downregulated miRNAs like miR-193b, miR-215-3p, miR-217, and miR-768-3p are linked with metastasis-preventing mechanisms as TS miRNAs (Table 2).

The reviewed literature shows correlations between changes in miRNA expression and brain metastasis in lung cancer. Most studies also indicate that these dysregulated miRNAs are indeed linked to well-known mechanisms of metastasis, including migration, invasion, proliferation, viability, self-renewal, and angiogenesis, using in vitro evidence. These studies’ findings suggest a potential role of those miRNAs in promoting brain metastasis. However, it is important to note that even when in vitro experiments show that specific miRNAs are involved in regulating mechanisms associated with metastasis, this evidence does not necessarily prove that the presence or absence of these miRNAs is required for the metastasis to occur in the brain. Further experiments, such as in vivo models, are essential to confirm whether these miRNAs play a causal role in brain metastasis outcomes in lung cancer. Table 2 displays those dysregulated miRNAs associated with brain metastasis that act as oncomiRs or TS miRNAs based on in vitro evidence. However, they require further experimental evidence directly supporting their promoting or inhibiting effect on brain metastasis outcomes.

For instance, levels of miR-21 are upregulated in the serum of NSCLC patients with brain metastasis. Overexpressing miR-21 in NSCLC cell lines increases cell migration, invasion, proliferation, and endothelial cell tube formation. However, no additional experimental evidence supports promoting brain metastasis [70]. In a separate study, the induced overexpression of miR-21 caused NSCLC patient-derived stem cell lines with brain metastasis (BMICs) to improve their ability to renew and proliferate. The study demonstrated that STAT3 interacts with the miR-21 promoter. Knocking down STAT3 decreased the self-renewal of BMIC, reduced intracranial tumor size in a mouse model, and affected predicted targets of miR-21. This supports the idea that STAT3 regulates the metastatic behavior of stem cells by activating miR-21. However, it is still unclear whether miR-21 directly contributes to the development of brain metastasis [78]. Another study found that miR-328 was upregulated in NSCLC tissue with brain metastasis. The overexpression of miR-328 promotes cell migration, suggesting a potential oncogenic role. However, its specific role in promoting brain metastasis has yet to be explored [79]. Another miRNA, miR-378, was upregulated in lung and brain tissue of NSCLC patients with brain metastasis. The overexpression of miR-378 enhances cell survival, migration, and invasion of lung cancer cells, as well as tumor growth and vasculogenic mimicry. Nonetheless, experiments related to brain metastasis promotion have not yet been conducted [80].

On the other hand, miRNAs such as miR-193b and miR-217 are downregulated in brain metastatic lung cell lines. Their induced overexpression inhibits migration and invasion of NSCLC cells, indicating a tumor suppressor role [82,84]. Another downregulated miRNA, miR-215, is downregulated in metastatic brain tissue from NSCLC patients. Its induced overexpression reduces NSCLC migration and invasion by targeting leptin and SLC2A5, indicating its tumor suppressor role. This study also found that lnc-REG3G-3-1, highly expressed in brain metastasis tissues, targets miR-215 and increases subcutaneous tumor size in vivo. However, whether miR-215 is required to promote brain metastasis was not explored [83].

Several studies establish that the brain’s microenvironment significantly impacts the metastatic behavior of cancer cells, including their miRNA expression. Specifically, in the case of lung cancer, co-culturing SCC lung cancer cells with astrocytes results in a reduction in the expression of miRNA-768-3p. This reduction contributes to enhanced cell viability and amplified K-ras expression in tumor cells, indicating a tumor suppressor role of miR-768-3p. Observations demonstrated a decrease in miRNA-768-3p levels in patients’ brain metastases compared to normal brain tissue and primary tumors. Nevertheless, the precise role of miR-768-3p in driving brain metastasis remains to be conclusively determined [85]. Another study found that radiation-induced M1-type microglia increases both endogenous and secreted levels of miR-9. The supernatant of irradiated M1-type microglia and the overexpression of miR-9 in A549 cells inhibits the mesenchymal–epithelial transition (MET) of A549 cells. While low-dose irradiation reduced brain metastasis of A549 cells in mouse brain models, the study did not provide experimental evidence of the miR-9 role in brain colonization [77].

### 4.3. miRNAs Involved in Crossing the BBB

**Figure 1:**
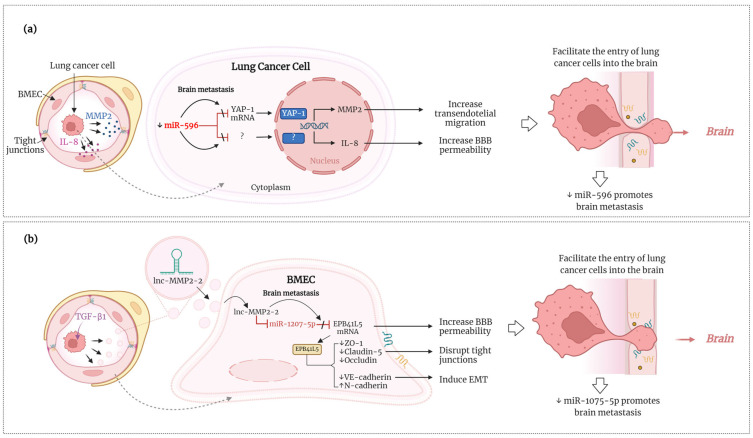
Figure depicting the proposed mechanism of action of (**a**) miR-596-3p [86] and (**b**) miRNA-1207-5p [89], which contribute to the spread of lung cancer to the brain by disrupting the BBB. A more detailed description is provided in the main body of the text. ↓, downregulation; BMEC, brain microvascular endothelial cells; lnc, long non-coding RNA. This figure was created using BioRender.com.

Various studies have provided strong experimental evidence that miRNAs are required for lung cancer cells to spread to the brain, based on both in vitro and in vivo evidence. These studies’ findings support that miRNAs play a causal role in brain metastasis in lung cancer. These specific miRNAs and the molecular mechanism they regulate could be feasible potential targets for future therapy. Section 4.3 and Section 4.4 highlight miRNAs experimentally linked to mechanisms that promote or inhibit brain metastasis, supported by evidence from in vitro and in vivo models (Table 3). MiR-596-3p and miR-1207-5p are two miRNAs found in lower levels in NSCLC brain metastases. Their reduced expression contributes to the spread of cancer to the brain by disrupting the BBB (Table 3 and Figure 1). MiR-596 is downregulated in brain metastatic lesions of NSCLC patients and brain metastatic lung cancer cells compared to the primary NSCLC tissues and parenteral cells, respectively. When miR-596-3p is overexpressed, it inhibits transendothelial cancer cell migration by blocking YAP1-induced MMP2 expression. It also reduces BBB permeability by suppressing the secretion of IL-8. Significantly, the overexpression of miR-596-3p hinders brain metastasis of cancer stem cell-derived brain metastasis in vivo models, confirming that miR-596-3p critically inhibits the capacity of NSCLC cells to metastasize to the brain (Figure 1a) [86].

**Table 3:** MicroRNAs that regulate mechanisms associated with brain metastases in lung cancer.

MicroRNA	Expression	Source	Effect	Function	Step of the Brain Metastasis Process	Reference
miR-95-3p	Down	Brain metastatic AD cancer cells. Brain metastatic tissues from lung cancer patients	Induced overexpression inhibits brain metastasis, cell proliferation, and invasiveness, via targeting Cyclin D1	TS miRNA	Brain colonization. Cell proliferation and invasiveness.	[87]
miR-145-5p	Down	NSCLC brain metastatic tissue.	Induced overexpression of miR-145-5p restrains brain orthotopic tumor engraftment and impairs cell migration.	TS miRNA	Cell migration	[88]
miR-596-3p	Down	NSCLC brain metastatic tissue. Brain metastatic lung cancer cells.	Downregulation promotes brain metastasis of NSCLC cells by modulating YAP1 and IL-8.	TS miRNA	Transendothelial migration. Alteration of BBB permeability.	[86]
miR-1207-5p	Down	BMECs	Downregulation promotes brain metastasis of NSCLC cells via destruction of tight junctions and increased permeability in BEMCs, through the TGF-β1-lnc-MMP2-2-miRNA-1207-5p/EPB41L5 axis.	TS miRNA	Disruption of BBB	[89]
miR-4317	Down	NSCLC tissue and serum	Induced expression inhibited proliferation, migration, and invasion of NSCLC cells by targeting FGF9 and CCND2. miR-4270 knockdown increases brain metastasis of GLC82 cells.	TS miRNA. Survival predictor biomarker in serum.	Cell proliferation, migration, and invasion.	[90]
miR-330-3p	Up	Serum of NSCLC with brain metastasis	Promotes proliferation, migration, and EMT of NSCLC cells. Promotes brain metastasis via orthotopic implantation directly into the brain.	OncomiR. Predictive biomarker in serum.	Cell proliferation, migration, and EMT.	[76]
miR-423-5p	Up	Lung AD tissue from patients with brain metastasis.	Promotes colony formation, cell motility, migration, and invasion of AD cells in vitro. Increases tumor burden and distant brain metastasis in vivo.	OncomiR.	Brain colonization. Cell invasion.	[91]
Exosomal miR-142-3p	“Abundant”	Astrocytes	Astrocytes disrupts brain metastasis of AD cells by mediating the downregulation of TRPA1 through exosome-delivered miRNA-142-3p	TS miRNA	Brain colonization. Invasion, and proliferation.	[92]

TS, tumor suppressor; NSCLC, non-small cell lung cancer; BMECs, brain microvascular endothelial cells; YAP1, yes-associated protein 1; AD, lung adenocarcinoma; EMT, epithelial–mesenchymal transition; MET, mesenchymal–epithelial transition; FGF9, fibroblast growth factor 9; CCND2, cyclin D2; TRPA1, transient receptor potential A1; BM, brain metastasis.

Other cell lineages, in addition to cancer cells, also serve as the source of miRNAs associated with brain metastasis, such as miR-1207-5p in BEMCs, showing the relevance of cancer cells’ interactions with the brain microenvironment. Researchers found that the long non-coding RNA, lnc-MMP2-2, is abundant in exosomes derived from TGF-β1-stimulated A549. The lnc-MMP2-2 found in TGF-β1 exosomes suppresses the expression of miR-1207-5p in BMECs, which leads to the increased production of its target protein, erythrocyte membrane protein band 4.1 like 5 (EPB41L5). When lnc-MMP2-2 and EPB41L5 are overexpressed in BMECs, it promotes markers of EMT, disrupts tight junctions, and increases the permeability of the BMEC monolayer in vitro. Furthermore, the overexpression of miR-1207-5p in BMECs leads to increased expression of tight junction proteins and reduces BMECs’ monolayer permeability, indicating the miRNA’s regulatory function. These changes result in a higher permeability of BBB, facilitating the entry of lung cancer cells into the brain and promoting brain metastasis in vivo (Figure 1b) [89].

### 4.4. miRNAs Involved in Brain Colonization

**Figure 2:**
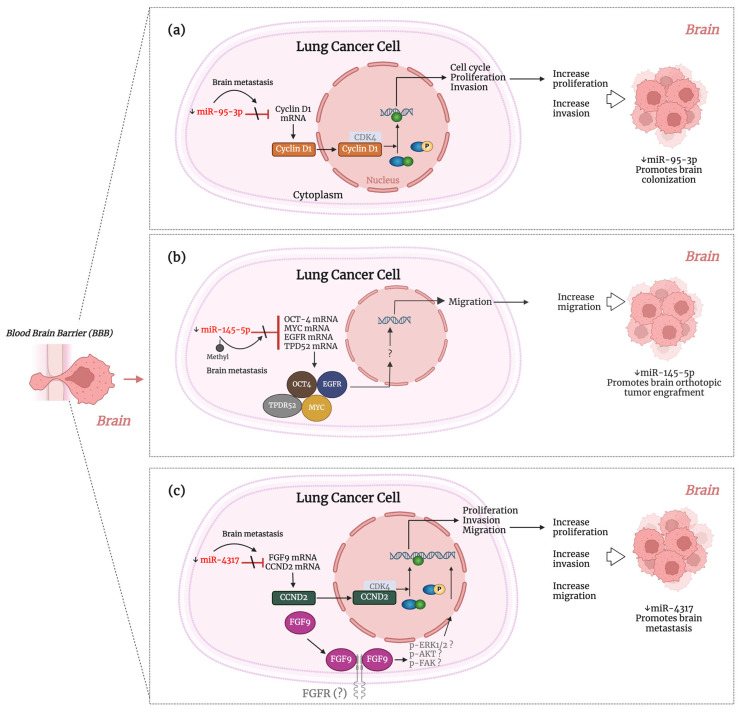
Figure depicting the proposed mechanism of action of miRNAs involved in brain colonization as TS miRNAs (**a**) miR-95-3p [87] and (**b**) miRNA-145-5p [88], (**c**) miRNA-4317 [90], which contribute to the spread of lung cancer to the brain by regulating proliferation, migration, and invasion of lung cancer cells. The molecular elements not experimentally tested in each study are shown in gray. A more detailed description is provided in the main body of the text. ↓, downregulation. This figure was created using BioRender.com.

Several studies have highlighted the pivotal role of miRNAs in the spread of lung cancer into the brain. These brain metastasis-related miRNAs regulate lung cancer cells’ migration, invasion, proliferation, and EMT capabilities, ultimately influencing the outcome of brain colonization (Table 3). MiRNAs such as miR-95-3p, miR-145, and miR-4317 are downregulated in NSCLC with brain metastasis and regulate cell proliferation, migration, and invasion of lung cancer cells. These three miRNAs act as TS miRNAs in brain metastasis. For instance, levels of miR-95-3p are reduced in NSCLC brain metastatic tissue and brain metastatic lung adenocarcinoma (AD) cells. The overexpression of miR-95 minimizes the spread of brain metastatic AD cells to the brain in vivo. Mechanistically, miR-95 inhibits cyclin D1 expression, suppressing proliferation invasion and clonogenicity of brain metastatic AD cells (Figure 2a) [87].

Similarly, levels of miR-145 are diminished in NSCLC brain metastatic tissue compared to the primary tumor. This downregulation is due to the methylation of miR-145′s promoter. When miR-145 is overexpressed in tumor cells, it restricts brain orthotopic tumor engraftment and impairs lung cancer cell migration in vitro. This study showed that miR-145-5p targets the proteins associated with oncogenesis OCT-4, EGFR, MUC-1, c-MYC, and TPD52 in lung cancer cells. However, the significance of these proteins in the spread to the brain still requires further experimental investigation (Figure 2b) [88]. MiR-4317 was discovered to be downregulated in the tissue and serum of NSCLC patients. Researchers found a correlation to brain metastasis by knocking down miR-4317 in GLC82 cells, leading to a significant increase in brain metastasis in vivo. When miR-4317 is overexpressed, it downregulates proliferation, colony formation, migration, and invasion of A973 and A549 lung cancer cells. In this study, miR-4317 targets fibroblast growth factor 9 (FGF9) and cyclin D2 (CCND2), affecting the migration and invasion of lung cancer cells (Figure 2c). In addition, low miR-4317 expression levels in NSCLC patient serum were identified as an independent prognostic factor for poor overall survival (OS) [90].

**Figure 3:**
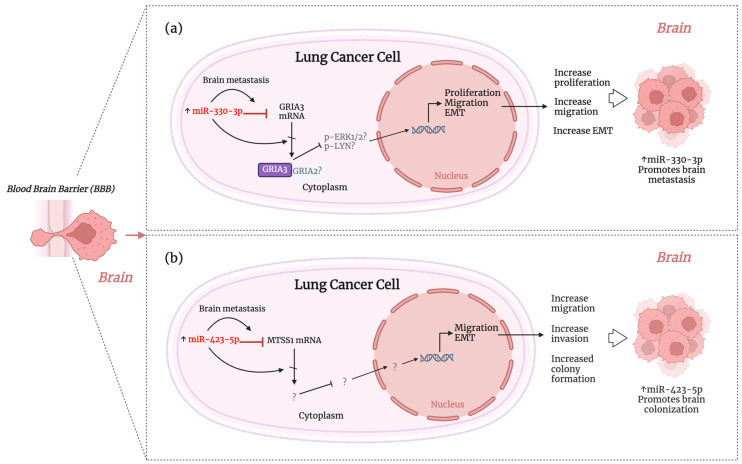
Figure depicting the proposed mechanism of action of miRNAs involved in brain colonization as OncomiRs, (**a**) miR-330-3p [76] and (**b**) miRNA-423-5p [91], which contribute to the spread of lung cancer to the brain by regulating proliferation, migration, EMT, and colony formation of lung cancer cells. A more detailed description is provided in the main body of the text. ↑, Upregulation. This figure was created using BioRender.com.

On the other hand, miR-330-3p and miR-423-5p were found to be upregulated in the serum and lung tissue of NSCLC with brain metastasis, respectively, acting as oncomiRs (Table 3 and Figure 3). MiR-330-3p promotes the proliferation, migration, and EMT of NSCLC cells in vitro and brain metastasis via orthotopic implantation directly into the brain in vivo. miR-330-3p targets the glutamate ionotropic receptor AMPA type subunit 3 (GRIA3). The overexpression of GRIA3 decreased the migration, invasiveness, and TGF-β1-induced EMT in NSCLC cells, suggesting its potential implication in metastasis (Figure 3a) [76]. Moreover, Kaplan–Meier analysis showed a shorter time for developing brain metastasis with higher levels of miR-330-3p [76], suggesting a role as a potential noninvasive biomarker. Another study found that miR-423-5p increases tumor burden and distant brain metastasis of NSCLC cells in vivo. This study showed that miR-423-5p targets the metastasis of the suppressor 1 (MTSS1). MTSS1 exhibited negative or low expression in AD tissue with brain metastasis, which was associated with poor survival. However, the role of MTSS1 in the mechanism of brain metastasis was not investigated further (Figure 3b) [91].

The following section summarizes the exosome-derived miRNAs associated with brain metastasis in lung cancer as reviewed in the literature.

### 4.5. Exosomal miRNAs in Lung Cancer Brain Metastasis

**Figure 4:**
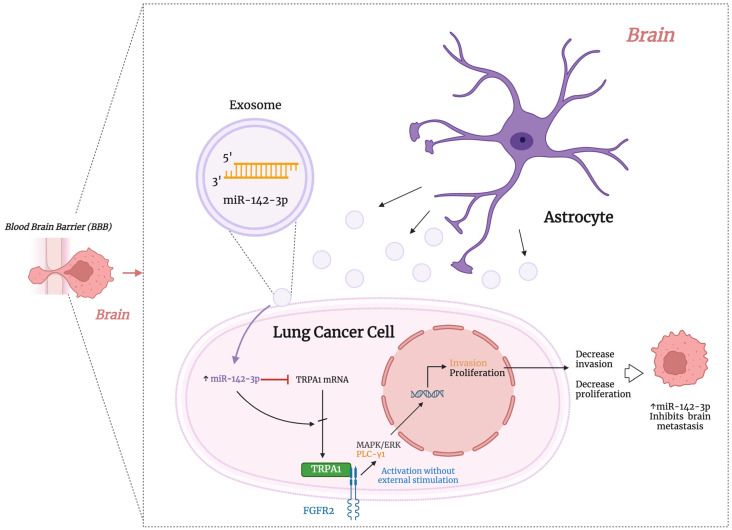
Figure depicting the proposed action mechanism of exosomal miRNAs I miR-142-3p [92] involved in brain metastasis as TS miRNA. A more detailed description is provided in the main body of the text. ↑, upregulation. This figure was created using BioRender.com.

According to a recent study, patients with lung cancer who have brain metastases have upregulated levels of miR-550a-3-5p in their exosomes from plasma than patients who do not. When BMECs were treated with those exosomes, the treatment decreased viability and migration, increased cell apoptosis, and reduced cells in the G0/G1 phase. Similar effects were observed when BEMCs were transfected with miR-550a-3-5p mimics, while the opposite effects were seen with miR-550a-3-5p inhibitors. miR-550a-5-5p directly targets the Yes1-associated transcriptional regulator (YAP1). YAP1 is a transcription regulator that promotes the transcription of genes associated with cell proliferation, apoptosis, homeostasis, and DNA repair. These findings indicate that exosomal miR-550a-3-5p regulates cell viability, apoptosis, cell cycle, and migration of brain endothelial cells through YAP1. However, further research is needed to understand the effects of lung cancer-associated brain metastasis (Table 2) [81]. Another study discovered that when lung adenocarcinoma cells encounter astrocytes, the expression of an ion channel called transient receptor potential ankyrin-1 (TRPA1) is decreased. This decrease occurs because brain astrocytes release exosomal miRNA-142-3p, which then targets and reduces TRPA1 levels in the cancer cells. The reduction in TRPA1 levels stops the activation of the fibroblast growth factor receptor 2 (FGFR2), preventing the spread of cancer cells to the brain. This process was observed in both in vitro and in vivo models. Mechanistically, TRPA1 binds to the proline-rich area of FGFR2, leading to FGFR2 activation independently of external stimulation. The binding of TRPA1 and FGFR2 increases the activation of MAPK/ERK and PLC-γ1 pathways, leading to the proliferation and invasion of cancer cells. These findings highlight astrocytes’ crucial role in preventing lung cancer cells’ metastasis to the brain and underscore the role of TRPA1 in controlling FGFR2-driven metastasis (see Figure 4) [92].

### 4.6. miRNAs with Targeting Potential for Brain Metastasis Therapy in Lung Cancer

**Figure 5:**
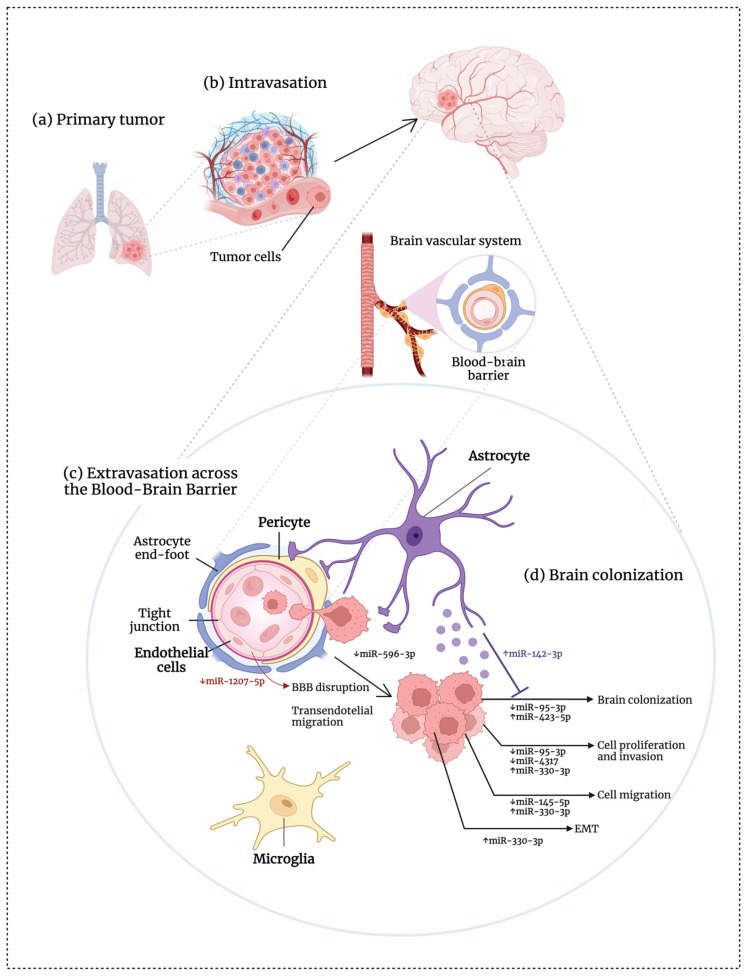
Summary of the role of miRNAs in regulating mechanisms associated with brain metastases in lung cancer. (**a**) Tumor cell migration from the primary site; (**b**) intravasation into the bloodstream; (**c**) extravasation across the blood–brain barrier; (**d**) brain colonization. ↑, upregulation; ↓, downregulation; BBB, blood–brain barrier. This figure was created using BioRender.com.

Figure 5 summarizes the role of miRNAs in regulating the mechanisms associated with brain metastases in lung cancer, supported by evidence from in vitro and in vivo models. As previously stated, the findings of these studies provide strong evidence that these specific miRNAs play a causal role in the spread of lung cancer cells to the brain. As a result, these miRNAs could be potential targets for future therapy. In this context, Table 3 and Figure 1, Figure 2, Figure 3 and Figure 4 provide descriptions of the miRNAs with targeting potential.

**Figure 6:**
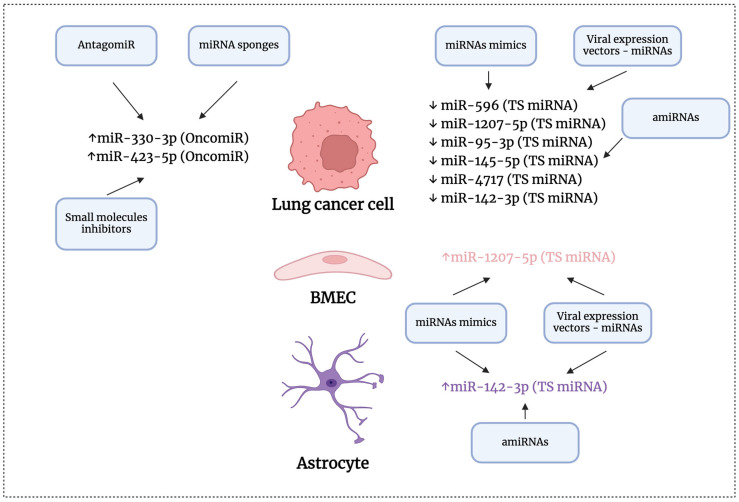
A diagram showing potential strategies for targeting miRNAs to inhibit brain metastasis in lung cancer. ↑, upregulation; ↓, downregulation; BMECs, brain microvascular endothelial cells. A more detailed description is provided in the main body of the text. This figure was created using BioRender.com.

Different strategies are being developed for miRNA therapeutics to counteract the harmful effects of abnormally expressed miRNAs in several diseases. Other publications have extensively reviewed these strategies, such as in the following references [93,94]. Figure 6 illustrates the potential approaches for targeting specific miRNAs to inhibit brain metastasis in lung cancer. In summary, synthetic miRNAs (miRNA mimics) and expression vectors containing miRNA encoding sequences are utilized to restore miRNA levels. Additionally, artificially designed miRNA constructs (amiRNAs), which combine siRNA sequences and scaffolds of primary miRNA transcripts, are used for the same purpose. Furthermore, oligonucleotides that inhibit miRNAs (anti-miRs or antagomiRs) and small cell-permeable compounds that impede miRNA biogenesis or bind to miRNA-specific secondary structures are employed to reduce miRNA levels. On the other hand, miRNA sponges, such as circular RNAs, are designed with multiple miRNA binding sites and function by sequestering several endogenous miRNAs.

We found that no current miRNA-based lung cancer brain metastasis therapies are in progress in pre-clinical or clinical trials. This field—miRNAs in brain metastasis from lung cancer—is in the discovery stage [93,94,95].

## 5. Conclusions

Brain metastasis poses a significant clinical challenge for advanced lung cancer patients. The high incidence of brain metastases significantly impacts prognosis, available treatments, and overall quality of life for these patients. Understanding the genetic and molecular mechanisms underlying brain metastases is crucial for enhancing patient outcomes.

Research has shown that miRNAs represent a pivotal component in the molecular landscape of brain metastasis from lung cancer. They play a critical role in this complex process by acting as key regulatory molecules in gene expression. The reviewed studies highlight the potential dual role of miRNAs. Specific miRNAs can promote or suppress brain metastasis by targeting multiple signaling pathways and molecular mechanisms. They are involved in various stages of brain metastasis, such as transendothelial migration and dysregulation of the BBB, EMT, proliferation, invasiveness, and brain colonization. Evidence indicates that specific miRNAs are not only linked to the presence of brain metastasis from lung cancer but are also necessary for metastasizing to the brain. MiRNAs downregulated in tumor cells, such as miR-95-3p, miR-145-5p, miR-596-3p, and miR-4317, promote brain metastasis in vivo models. In addition, miRNAs upregulated in tumor cells, such as miR-330-3p and miR-423-5p, promote brain metastasis in vivo. Other cell lineages also serve as the source of miRNAs associated with brain metastasis, such as miR-1207-5p in BMECs and miR-142-3p in astrocytes, showing the relevance of cancer cells’ interactions with the brain microenvironment. These miRNA-mediated regulation findings not only provide valuable insights into the metastatic process but may hold the promise to identify potential therapeutic targets for preventing and treating brain metastases.

The reviewed literature also indicated that further experimental investigation is required to confirm that several miRNAs that function as oncomiRs or TS miRNAs have a causal effect in the mechanisms of spreading to the brain (Table 2). This highly valuable information should be added to the field of knowledge. Moreover, the literature indicates that specific miRNAs and signatures may be useful biomarkers for diagnosing and predicting brain metastasis in lung cancer patients. However, at this point in lung cancer investigations, it is essential to research larger patient cohorts to validate the role of miRNAs as reliable biomarkers for brain metastasis.

Although the field of miRNA therapeutics for brain metastasis in lung cancer is still in the early discovery stage, several strategies could be used to apply miRNA therapeutics (see Figure 6) for those miRNAs that experimental evidence suggests play a causal role in the spread of lung cancer cells to the brain (Table 3).

Future research on miRNAs in brain metastasis from lung cancer should focus on several key areas to unlock their potential in clinical applications. It is critical to verify the role of miRNAs in driving brain metastasis using in vivo models. This evidence will open up possibilities for therapeutics to prevent metastasis or decrease tumor burden. It is also relevant to conduct comprehensive profiling of miRNAs in larger patient cohorts to validate their role as reliable biomarkers and to identify novel miRNAs associated with brain metastasis. Furthermore, combining miRNA research with other analytical technologies, like proteome, genome, and metabolome analysis, will uncover multi-level regulatory networks and offer a thorough understanding of the metastatic process. This approach could reveal new therapeutic targets and enhance the development of multi-modal treatment strategies. Continued research in this field can improve the diagnosis, prognosis, and treatment of brain metastasis, ultimately improving patients’ survival and quality of life.

## Data Availability

No new data were created or analyzed in this study. Data sharing does not apply to this article.
